# RBM10 promotes transformation-associated processes in small cell lung cancer and is directly regulated by RBM5

**DOI:** 10.1371/journal.pone.0180258

**Published:** 2017-06-29

**Authors:** Julie J. Loiselle, Justin G. Roy, Leslie C. Sutherland

**Affiliations:** 1 Biomolecular Sciences Program, Laurentian University, Sudbury, Ontario, Canada; 2 Department of Chemistry and Biochemistry, Laurentian University, Sudbury, Ontario, Canada; 3 Health Sciences North Research Institute (HSNRI), Sudbury, Ontario, Canada; University of South Alabama Mitchell Cancer Institute, UNITED STATES

## Abstract

Lung cancers are the leading cause of cancer-related deaths worldwide, with small cell lung cancer (SCLC) being the most aggressive type. At the time of diagnosis, SCLC has usually already metastasized, and an astonishing 95% of patients eventually succumb to the disease. This highlights the need for more effective SCLC screening and treatment options. Interestingly, the earliest and most frequent genetic alteration associated with lung cancers involves a lesion in the region to which the RNA binding protein *RBM5* maps. We have recently shown that a decrease in *RBM5* expression may be a key step in SCLC development, as RBM5 regulated many transformation-associated processes in SCLC cells. RBM5 is structurally and functionally similar to another RNA binding protein, RBM10. Both proteins have tumor-suppressor properties in a variety of cancer cell lines, and it has been suggested that *RBM5* expression can influence *RBM10*. Due to their similarities, and the recent evidence that *RBM10* is mutated in up to 21% of lung cancers, we hypothesized that RBM10 would share RBM5’s tumor-suppressor properties in SCLC. Using transcriptome analysis and functional assays, we show, however, that RBM10’s function was opposite to what we hypothesized; in the endogenously *RBM5*-null GLC20 SCLC cell line, RBM10 actually promoted cell proliferation and other transformation-associated processes. Using RNA immunoprecipitation followed by next generation sequencing (RIP-Seq) and Western blotting, we demonstrate that RBM5 post-transcriptionally regulated *RBM10* expression *via* direct interaction with specific *RBM10* splice variants. We propose a working model describing the impact of this interaction on cellular processes. Our results provide evidence that *RBM10* expression, in *RBM5*-null tumors, may contribute to tumor growth and metastasis. Measurement of both *RBM10* and *RBM5* expression in clinical samples may therefore hold prognostic and/or potentially predictive value.

## Introduction

According to the World Health Organization, cancer is one of the main causes of morbidity and mortality worldwide. Lung cancers are not only one of the most diagnosed cancers, but also the main cause of these cancer-related deaths, claiming over 1.6 million lives in 2012 [1]. The most aggressive form of lung cancer is small cell lung cancer (SCLC) [2]. In 90% of SCLC cases, the disease has already metastasized when the patient is diagnosed, which limits treatment options [3]. An astonishing 95% of SCLC patients eventually succumb to the disease, highlighting the need for more effective detection and treatment options [4]. Interestingly, the earliest and most frequent genetic alteration that occurs in lung cancer involves deletion within the 3p21.3 region [5–7]. The RNA binding protein, *RBM5*, resides within this region and is significantly downregulated [8], but not deleted [6], in the majority of lung cancers. Recently, our group demonstrated that this decline in *RBM5* expression may be a key step in the establishment of the transformed state of lung cells; RBM5 is responsible for slowing the cell cycle, promoting apoptosis, and downregulating transformation-associated processes such as angiogenesis in SCLC cells [9]. *RBM5* may therefore be an important marker for SCLC risk, and could guide the development of more effective screening and/or treatment options.

**Fig 1:**
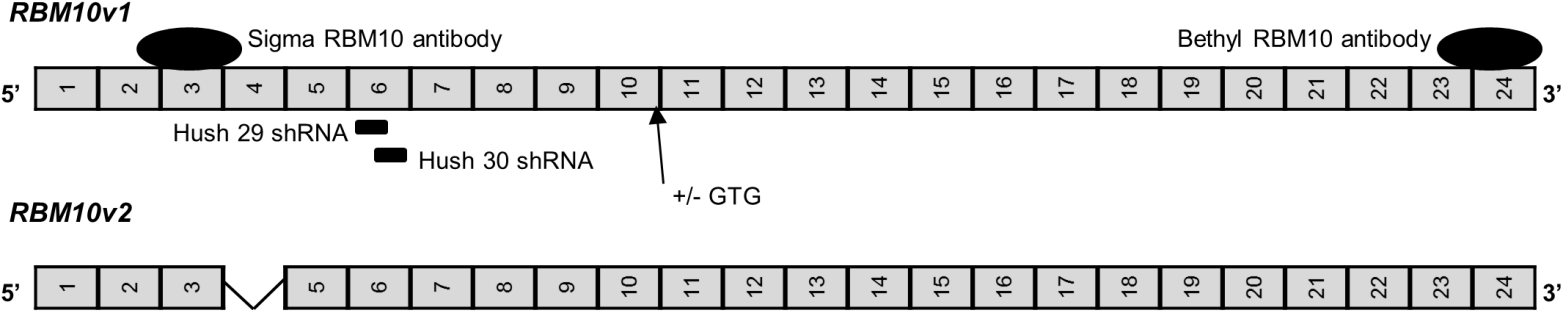
Schematic representation of RBM10v1/v2 exons. Exons are represented by grey boxes outlined in black. Box sizes are not representative of actual exon size. Black ovals represent approximate location of the epitopes recognized by the antibodies used in this study. Solid black lines represent the approximate location of shRNA RBM10 targets, which were not variant specific. Corresponding names of antibodies and shRNA are listed next to their approximate location. Arrow indicates position of the GTG codon coding for the valine residue that can be differentially spliced from RBM10 transcripts (last amino acid coded by exon 10).

RBM5 is structurally similar to another RNA binding protein, RBM10. *RBM10* has two main alternative splice variants termed *RBM10* variant 1 (*RBM10v1*) and *RBM10* variant 2 (*RBM10v2*) [10–12]. Each main *RBM10* splice variant also codes for alternative isoforms with or without the addition of one valine residue (Fig 1) [13]. Structurally, RBM10v2 and RBM5 share 53% homology at the amino acid level [12].

RBM5 and RBM10 also share functional similarities, and both have tumor-suppressor properties in various cancer cell lines [10, 12, 14]. Functional data for RBM10 are limited, but in certain studies, RBM10 promoted apoptosis and/or decreased cell proliferation; (1) *RBM10* expression correlated with decreased cell proliferation and increased apoptosis in primary chondrocytes induced to hypertrophy [15], (2) *RBM10v1* expression in breast cancer specimens correlated with the expression of proapoptotic BAX and the tumor suppressor gene p53 [16], (3) stable *RBM10* knockdown (KD) in ovarian cancer cells (HeLa) correlated with a significant increase in colony formation by clonogenic assays [17], (4) in lymphoblastic leukemia (Jurkat) and breast cancer (MCF-7) cells, transient *RBM10* overexpression correlated with increased levels of apoptosis, whereas stable RBM10KD correlated with lower TNF-α protein levels, as well as decreased TNF-α mediated apoptosis [10], and (5) in a mouse xenograft tumor model with HeLa cells, RBM10KD enhanced tumor growth [14]. In contrast, other studies suggest an anti-apoptotic function for RBM10; (1) RBM10KD in SHSY5Y human neuronal cells augmented proapoptotic caspase activity after staurosporine exposure [18], (2) expression of both *RBM10* mRNA variants in breast cancer cells positively correlated with *VEGF* mRNA, a promoter of new blood vessel growth [16], and (3) in patients with metastatic melanoma, high *RBM10* expression correlated with increased disease aggressiveness [19]. Considered as a whole, these results suggest that the regulation of cell growth is an important aspect of RBM10 function, but that the mechanism regulating this function requires elucidation. Limited evidence suggests that this regulation depends, at least in part, on the related RNA binding protein RBM5 [20].

Considering the similarities between RBM5 and RBM10, and the recent finding that *RBM10* is mutated in up to 21% of invasive lung adenocarcinomas [21], we hypothesized that RBM10 shares RBM5’s tumor-suppressor properties in SCLC. Consequently, RBM10 may also hold prognostic potential for assessing SCLC risk and provide important information regarding the development and/or progression of this particularly aggressive form of lung cancer. To this end, we set out to determine RBM10’s role in SCLC, and compare it to that of RBM5.

## Materials and methods

### Cell culture & subline establishment

SCLC GLC20 cells were gifted by the late Dr. Charles Buys from the University of Groningen (Groningen, Netherlands) and cultured as previously described [9]. Stable *RBM5*-expressing T2 and C4 GLC20 sublines were established as previously described [9].

SCLC GLC20 *RBM10* stable KD sublines were generated from passage 7 GLC20 wild type cells. Several different dilutions of GLC20 cells were plated in 24-well flat-bottom plates (Sarstedt AG & Co., Nümbrecht, Germany) and incubated at 37°C with 5% CO_2_ in a humidified chamber for 24 hours. Half of the cells were transfected with a control shRNA vector “Hush 300” (TR30003, Origene Technologies, Inc, Rockville, U.S.A) and the other half with a 50:50 shRNA mix of “Hush 29” (TI308329, Origene, 5’-GCCTTCGTCGAGTTTAGTCACTTGCAGGA) and “Hush 30” (TI308330, Origene, 5’-AGTCACTTGCAGGACGCTACACGATGGAT). Hush 29 and 30 both target exon 6 of RBM10 and are not variant 1 or variant 2 specific (Fig 1). Cells were transfected using Lipofectamine 2000 (L2000) (Invitrogen, Life Technologies, Burlington, Canada). Transfected cells were selected using 0.1 μg/mL puromycin (Invitrogen) starting at 48 hours post-transfection. After seven days of selection, cells were treated with a Histopaque solution (Sigma-Aldrich) to separate live from dead cells. The stable negative control shRNA subline was named G300.3, and three surviving RBM10KD sublines were named G29/30.1, G29/30.3 and G29/30.4.

### RNA sequencing and analysis

RNA samples were isolated using Tri-Reagent (BioCan Scientific, Mississauga, Canada). All sequencing was performed by the Donnelly Sequencing Centre (Toronto, Canada) on the Illumina HiSeq 2500 platform. RNA-Seq library preparation and sequencing specifications were as previously described [9]. All specimens, therefore, had (1) quality score distributions over all sequences above 37 (on a phred 33 quality scale), as determined by FastQC (Babraham Bioinformatics, https://www.bioinformatics.babraham.ac.uk/projects/fastqc/), and (2) low quality ends trimmed during adapter trimming (quality cut-off of 26), as previous recommended [22]. Specifications for the GLC20 stable scramble control and RBM10KD (G29/30.4) were as follows; (1) RNA was not DNAse treated prior to sequencing, (2) reads were 125 bp long, and (3) both samples were multiplexed together and run in one lane. Sequencing data has been deposited to the NCBI Sequence Read Archive (SRA) database, under the SRA accession number (https://www.ncbi.nlm.nih.gov/Traces/study/) SRP106846, Bioproject PRJNA386251.

Sequencing results were analyzed and pathway analysis carried out as previously described [9].

### Western blotting

Performed as previously described [9]. Two rabbit anti-human RBM10 primary antibodies were also used: Sigma-Aldrich, HPA034972 and Bethyl Laboratories, A301-006A-1.

### RNA immunoprecipitation followed by next generation sequencing (RIP-Seq)

The basic RIP technique was carried out as previously described [23]. RNA was sequenced by the Donnelly Sequencing Centre on the Illumina HiSeq 2500 platform. Libraries were prepared using the Illumina TruSeq Stranded Total RNA library preparation kit with Ribozero Gold depletion and random priming. Associated samples were multiplexed together for sequencing, and each run was 125 bp and pair-ended. The RBM10 antibody used for RBM10 immunoprecipitation was from Sigma-Aldrich (catalogue number: HPA034972), and the non-specific IgG Control was from Cell Signaling (catalogue number: 2729). Sequencing data has been deposited to the NCBI Sequence Read Archive (SRA) database, under the SRA accession number (https://www.ncbi.nlm.nih.gov/Traces/study/) SRP106846, Bioproject PRJNA386251.

The FPKM and log2-fold change expression values generated by Cuffdiff were used to determine protein targets. The potential target had to meet the following inclusion criteria in the RBM5 or RBM10 RIP sample, as previously described [9]: (1) an FPKM value above one, and (2) a log2-fold change above one (and necessarily positive, indicating it was more highly expressed than in the Control RIP).

### Cell growth assay

GLC20 cells and RBM10-knockdowns were plated, as previously described [9], and monitored every day for five days by MTT assays. At the indicated time intervals, cells were treated with 10 μL of 5 mg/mL MTT (3-(4,5-dimethylthiazol-2-yl)-2,5-diphenyltetrazolium bromide) Reagent (Life Technologies) in PBS (Gibco) for 2 hours and 45 min at 37°C, as previously described [24]. Following incubation, cells were transferred to a 96-well Vee-bottom plate (Sarstedt) and subjected to centrifugation. Supernatant was discarded and the blue formazan precipitate was dissolved in DMSO (BDH Chemicals, VWR). Dissolved crystals were then transferred to a 96-well flat-bottom plate and, after a ten minute incubation, absorbances were read at 540 nm using a BioTek Synergy S4 Spectrophotometer (BioTek Instruments, Inc). Three biological replicates, using cells with different passage numbers, were performed.

Absorbances of each biological replicate were normalized to their respective day 0 absorbance value, and the averages of three or four biological replicates for each day are presented. A Two-way ANOVA statistical analysis was performed with the Bonferroni post-hoc test, comparing all growth to the pcDNA3 subline for the RBM5 sublines or G300.3 for the RBM10KD sublines, using Graphpad Prism 5 (Graphpad Software, Inc., San Diego, U.S.A).

## Results

### RBM10 RNA targets in GLC20 cells that express *RBM5* cDNA

**Fig 2:**
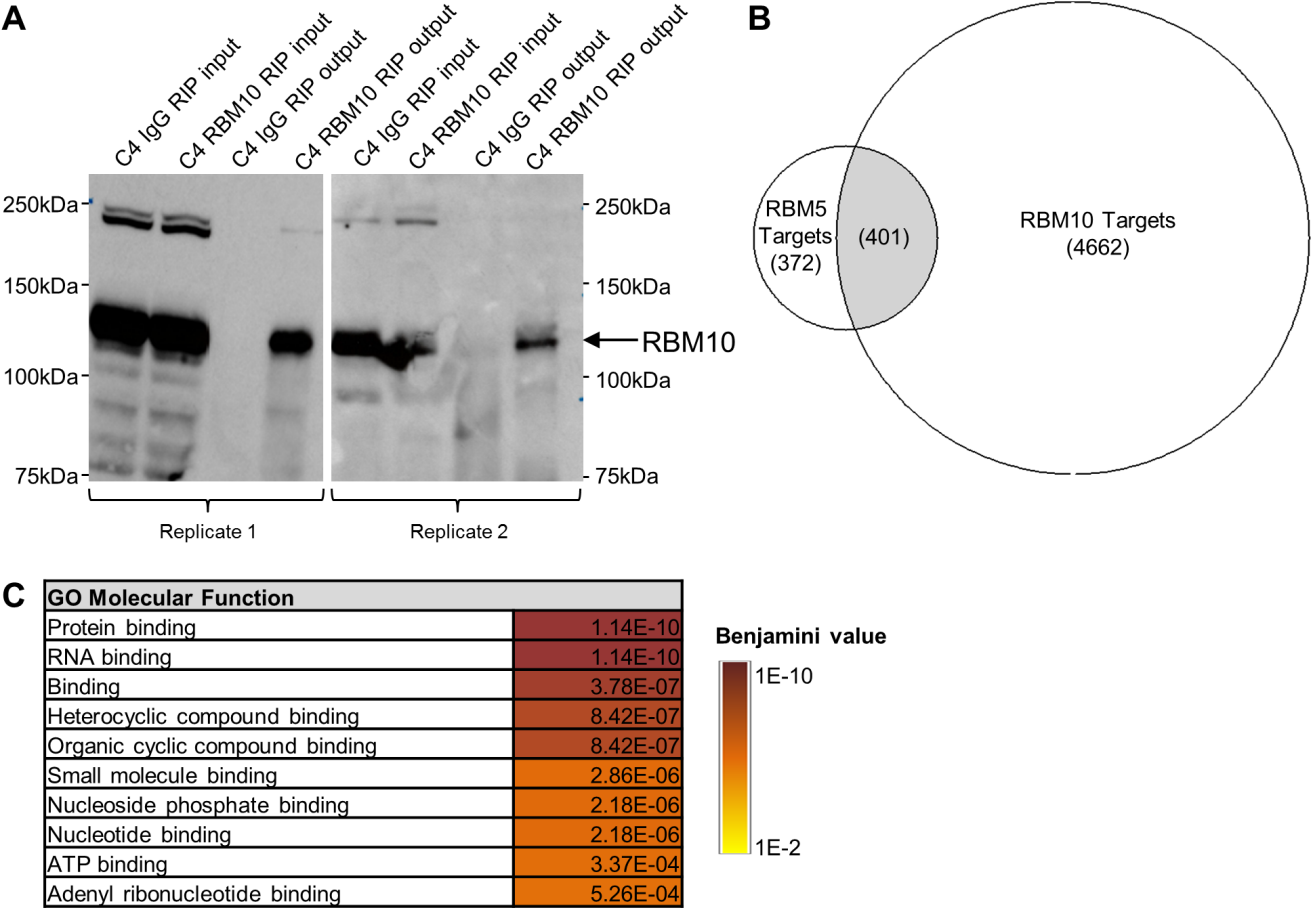
RBM10 RIP-Seq results and comparison to identified RBM5 targets. (A) Successful immunoprecipitation of RBM10 from C4 cells, demonstrated by Western blot of input and output protein samples. Blots probed for RBM10, using the same RBM10 antibody used in the immunoprecipitation experiment (Sigma-Aldrich). Control immunoprecipitation performed, using a non-specific rabbit IgG antibody. (B) Overlap between RBM5 and RBM10 targets in C4 GLC20 subline. (C) GO Molecular Function gene sets enriched in common RBM5 and RBM10 RIP-Seq targets, as determined by FIDEA. Values indicated are Benjamini values.

We first set out to compare RBM5 and RBM10 targets in SCLC. As RBM10 is an RNA binding protein, and was previously shown to be a part of prespliceosomal complexes A and B [25, 26], we determined that RNA Immunoprecipitation followed by next generation sequencing (RIP-Seq) was the most suitable method for identification of RBM10 targets. RIP-Seq involves antibody-driven immunoprecipitation of targeted endogenous protein without breakdown of protein-protein interactions, thus any RNA that is either directly bound to RBM10, and/or bound to RBM10 *via* another RBM10-bound protein would be captured. We anticipated that, having homology and similar functions, RBM10 targets would overlap with those of RBM5. Our group recently identified RBM5 targets using an endogenously RBM5-null SCLC cell line (S1 Table), GLC20 [24], from which three stable sublines—an empty vector control and two *RBM5* expressing (T2 and C4)–had been generated [9]. The “T2” subline was derived from a pool of transfected cells, and had *RBM5* expression levels comparable to non-tumor lung tissue, while the “C4” subline was derived from a single transfected cell, and had 6-fold higher levels of *RBM5* than the T2 subline [9]. As RBM5 targets were previously identified in the C4 GLC20 subline, RBM10 RIP-Seq experiments were also performed in this subline in order to directly compare RBM5 and RBM10 targets. Specifically, RBM10 RIP-Seq was performed in biological duplicate in C4 cells using a non-specific IgG as a Control IP and an anti-RBM10 antibody for RBM10-specific IP. Following confirmation of successful IP (Fig 2A), the associated RNA was sequenced (S1 Table).

Using moderate binding criteria, as previously described [9], we identified 5063 RBM10 targets. In our previous study, using the same RIP-Seq procedure and binding criteria, we had identified 773 RBM5 targets in this GLC20 subline [9]. RBM10, therefore, had many more targets than RBM5, suggesting the scope of RBM10’s influence in SCLC may be greater than that of RBM5.

Interestingly, more than half of RBM5’s targets were shared by RBM10 (Fig 2B), confirming that they do have overlapping targets. Furthermore, this suggests that RBM10 plays a key role in RBM5 regulated processes but not *visa versa*. To determine the functional relevance of these common targets, we performed pathway analyses using the Functional Interpretation of Differential Expression Analysis (FIDEA) Program, a program that can be used to analyze various types of differential expression data [27]. Two different databases were used with the FIDEA Program; (1) the Kyoto Encyclopedia of Genes and Genomes (KEGG) Database, which groups genes based on established molecular interactions [28, 29], and (2) the Gene Ontology’s (GO) Molecular Function Database, which groups genes based on cellular functions [30, 31]. Using a Benjamini value cut off of 0.005, no KEGG pathways were found to be enriched, a not unexpected result since multiple targets within a single signaling pathway would not necessarily be required for an effect. Using the same Benjamini value parameter, ten GO Molecular Function gene sets were enriched (Fig 2C). Interestingly, all enriched gene sets involved binding, including to ATP, nucleotides, protein, RNA and small molecules. Thus RBM5 and RBM10’s common targets may regulate a plethora of cellular processes, which may also explain why no individual KEGG pathways were enriched.

Since RBM10 had so many RNA targets that were not shared by RBM5 (Fig 2B), we went on to determine the functional importance of these RBM10-specific genes (will be referred to as RBM10-only targets). Using the FIDEA Program with the KEGG Database, we identified eight significantly enriched pathways (Table 1). The most enriched pathway was ‘Metabolic pathways’, consistent with the top RBM10 targets, which are all involved in different aspects of cell metabolism: NDUFA6 (oxidoreductase activity), MDP1 (phosphatase activity) and ATP6VIF (ion transfer). We were particularly interested to see that the ‘Oxidative phosphorylation’ pathway was the second most-enriched pathway. Notably, changes in oxidative phosphorylation are not only linked to metabolism (the top enriched pathway identified), but also the three disease states enriched in RBM10-only targets: Parkinson’s [32, 33], Huntington’s [34] and Alzheimer’s [35, 36]. The fact that five of the eight enriched KEGG pathways are intimately linked to oxidative phosphorylation strongly suggests that RBM10 plays a particularly important role in this process.

**Table 1:** Pathways and gene sets enriched in RBM10-only RIP-Seq targets.

Pathways & gene sets	Benjamini value
**KEGG**	
Metabolic pathways	1.78E-8
Oxidative phosphorylation	9.34E-5
Base excision pair	1.56E-4
Huntington’s disease	4.37E-4
Alzheimer’s disease	1.77E-3
Neurotrophin signaling pathway	1.79E-3
RNA transport	1.79E-3
Parkinson’s disease	2.46E-3
**GO Molecular Function**	
Protein binding	3.62E-34
Binding	6.15E-21
RNA binding	1.07E-13
Structural constituent of ribosome	1.63E-7
Catalytic activity	5.31E-7
Transcription cofactor activity	2.07E-7
Heterocycylic compound binding	2.07E-6
Organic cyclic compound binding	2.07E-6
Transcription factor binding transcription factor activity	4.98E-6
Transferase activity	3.29E-6
Protein binding transcription factor activity	4.34E-6
Enzyme binding	2.30E-6
Nucleic acid binding	2.30E-5
Kinase binding	2.30E-5
Transcription corepressor activity	3.61E-4
Protein kinase binding	1.19E-4
Mitogen-activated protein kinase binding	2.13E-3

Significantly enriched gene sets with Benjamini values below 0.005 are listed.

To determine if these RBM10-only targets shared similar functionality, we used the GO Molecular Function Database with the FIDEA Program. Enriched gene sets are listed in Table 1. Many of the enriched gene sets are still showing RBM10 targets involved in a plethora of binding activities, but a new functional activity involving transcription regulation emerged, suggesting that RBM10 has distinct RBM5-independent functions.

Taken together our RIP-Seq results show that although RBM5 and RBM10 share many common targets, both have distinct targets, with RBM10 having over six times more than RBM5. RBM5 and RBM10 targets, themselves, bind various cellular components, suggesting that RBM5 and RBM10 work together to regulate a variety of cellular processes in SCLC. On the other hand, targets bound by RBM10, but not RBM5, regulated oxidative phosphorylation and numerous aspects of gene expression and protein activity, suggesting RBM10 has distinct mechanisms of action that could be RBM5-independent.

### Effect of RBM10 inhibition on dehydrogenase activity in GLC20 cells

Our RIP-Seq results showed that RBM10 directly influenced metabolism, most specifically oxidative phosphorylation. Work in HeLa cells and primary chondrocytes had previously correlated RBM10 expression with decreased cell proliferation [15, 17]. Taken together, these observations suggested that *RBM10* expression would result in decreased SCLC cell proliferation and/or metabolic activity. We therefore chose to use the MTT assay to examine the functional significance of *RBM10* expression to SCLC.

**Fig 3:**
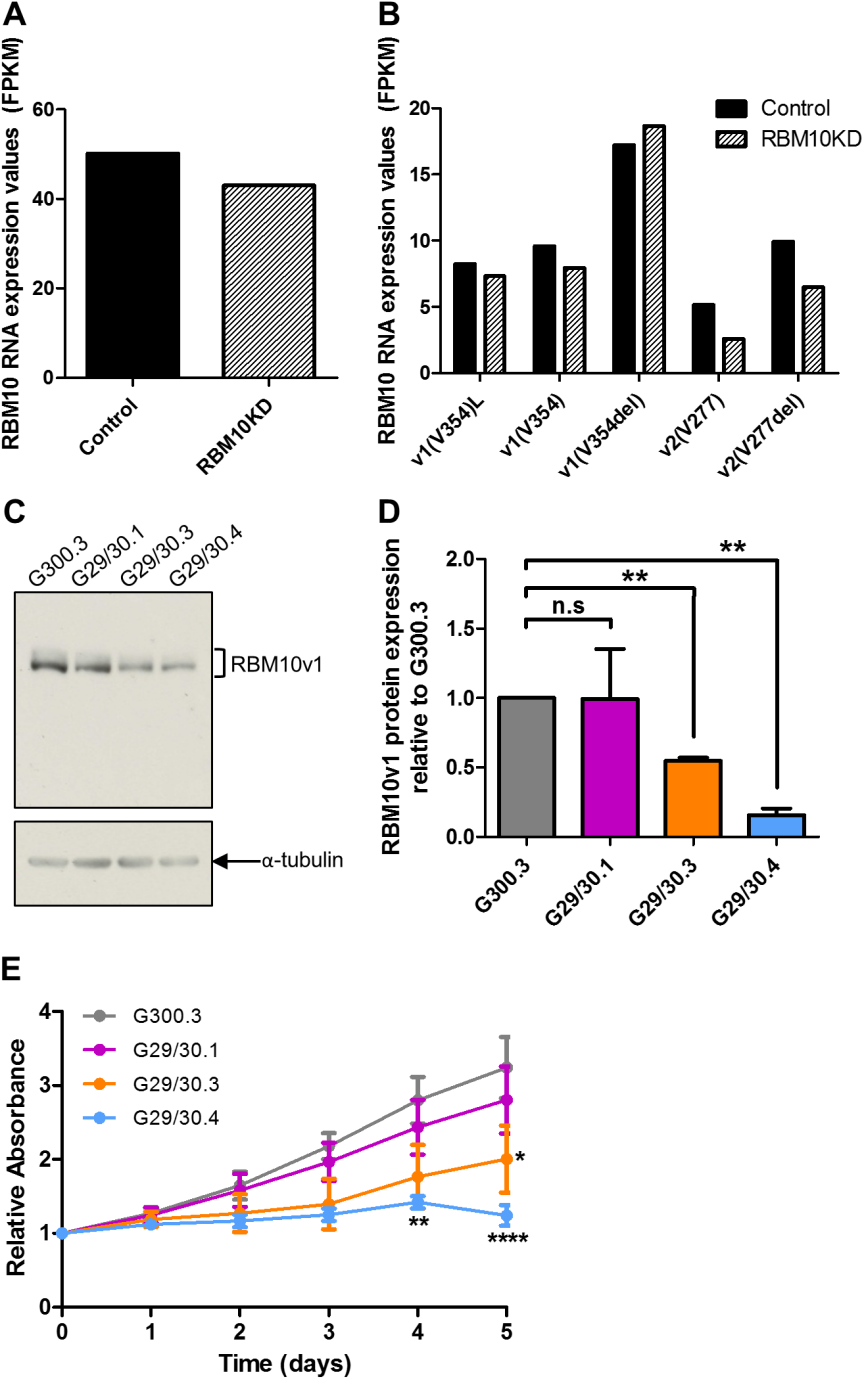
*RBM10* expression and dehydrogenase activity in control and stable *RBM10*KD GLC20 sublines. (A & B) *RBM10* mRNA levels, determined by RNA-Seq, for all *RBM10* variants combined (A) or for individual splice variants (B). ‘Control’ represents values from parental GLC20 cells and the control G300.3 GLC20 subline. RBM10KD sample used was the G29/30.4 subline, as it showed greatest decrease in RBM10 protein expression. (C & D) RBM10v1 protein levels were monitored in the G300.3, G29/30.1, G29/30.3 and G29/30.4 sublines using the Bethyl RBM10 antibody. (C) One representative Western blot result is presented for RBM10v1 and α-tubulin (loading control). (D) Densitometric results of the average RBM10v1 protein levels from three biological replicates performed in duplicate. Analysis was performed using the AlphaEaseFC, ‘1D-Multi’ analysis tool. Values of RBM10v1 were normalized to the α-tubulin of each biological replicate, and then made relative to the G300.3 control subline. Standard error is presented. Subline expression levels were compared using the Student’s unpaired *t*-test, between sublines. (E) G300.3, G29/30.1, G29/30.3 and G29/30.4 were grown for five days and dehydrogenase activity was monitored daily using an MTT assay. Absorbance was plotted relative to day 0. The average of three biological replicates performed in eight technical replicates with standard error is presented. A Two-way ANOVA was performed between the G300.3 and other sublines, with Bonferroni post-hoc analysis. * *p* < 0.05, ** *p* < 0.01, and **** *p* < 0.0001.

As a first step, we generated stable *RBM10* knockdowns (KDs) in the parental *RBM5*-null GLC20 cells. It is important to note that this GLC20 SCLC cell line is a particularly appropriate model for aggressive SCLC, as *RBM5* is downregulated in the majority of lung cancers [8]. RBM10KD was not performed in the T2 or C4 sublines since all *RBM5* expressed in these sublines results from exogenously introduced *RBM5* cDNA. These sublines, therefore, do not express any of the various *RBM5* splice variants that are present in endogenously *RBM5*-expressing systems, rendering results from RBM10KD experiments in these sublines non-physiological. As noted in Materials and Methods, GLC20 cells were subjected to transfection with two RBM10KD shRNA plasmids, together termed 29/30, that targeted an exon common to both *RBM10v1* and *RBM10v2*. Thus, knockdown of *RBM10v1* was not specifically targeted in this study, but due to the almost exclusive expression of RBM10v1 in the GLC20 cells at the protein level (Fig 3C), all decreases in RBM10 protein were reported as RBM10v1. Three stable sublines derived from three distinct pooled RBM10KD populations were produced, termed G29/30.1, G29/30.3 and G29/30.4, as well as one control subline, termed G300.3. Somewhat surprisingly, but in line with one of the known mechanisms of shRNA regulation [37, 38], RNA levels of *RBM10* were not diminished in any of the KDs (Fig 3A and 3B). At the protein level, although the G29/30.1 subline demonstrated great variance in protein expression, with no significant decrease in RBM10v1 expression relative to Control (99% expression, p = 0.9836), both G29/30.3 and G29/30.4 had significantly lower RBM10v1 protein levels relative to G300/3, with 55% (*p* = 0.0026) and 16% (*p* = 0.0033) the level of Control cells, respectively (Fig 3C and 3D).

The effect of RBM10KD on dehydrogenase activity was analyzed by MTT assay over a five day period (Fig 3E). The G300.3 and G29/30.1 sublines had similar dehydrogenase activity, a result that was anticipated as both sublines had nearly identical levels of RBM10v1 protein expression (Fig 3D). The G29/30.3 and G29/30.4 sublines, however, both displayed significantly reduced activity, relative to the G300.3 control. G29/30.4 had the greatest reduction in dehydrogenase activity, with significant differences on day four and day five (*p* < 0.01 and *p* < 0.0001, respectively), compared to G300.3.

These results indicate that the effect of RBM10 was level-dependent. More importantly, however, is the observation that inhibition of *RBM10* expression correlated with dehydrogenase activity that was decreased. Decreased activity could have resulted from either a decrease in cell number and/or a decrease in the metabolic rate of existing cells. Since this finding in an *RBM5*-null background is in contradiction to the previous work in *RBM5*-retaining HeLa cells [14, 17], our results suggest that RBM10 function can not only be altered, but actually reversed by RBM5.

### Effect of RBM10 inhibition on signaling pathways in GLC20 cells

To gain a more comprehensive understanding of the extent of RBM10’s influence in SCLC, we carried out RNA-Seq, because the MTT assay used above identifies only one functional consequence resulting from inhibition of RBM10. For these experiments, the stable RBM10KD G29/30.4 subline was used, as it had the greatest RBM10KD (referred to simply as RBM10KD in the following figures and the Discussion). As our Control, we sequenced and combined data for the stable pcDNA3 transfected GLC20 cells [9] and the stable scramble shRNA control G300.3. Sequencing and analysis were performed as previously described [9]. Specific parameters are listed in Materials and Methods. Using the Cufflinks suite, we identified 1157 significantly differentially expressed genes, representing approximately 4.45% of the transcriptome (S2 Table).

**Fig 4:**
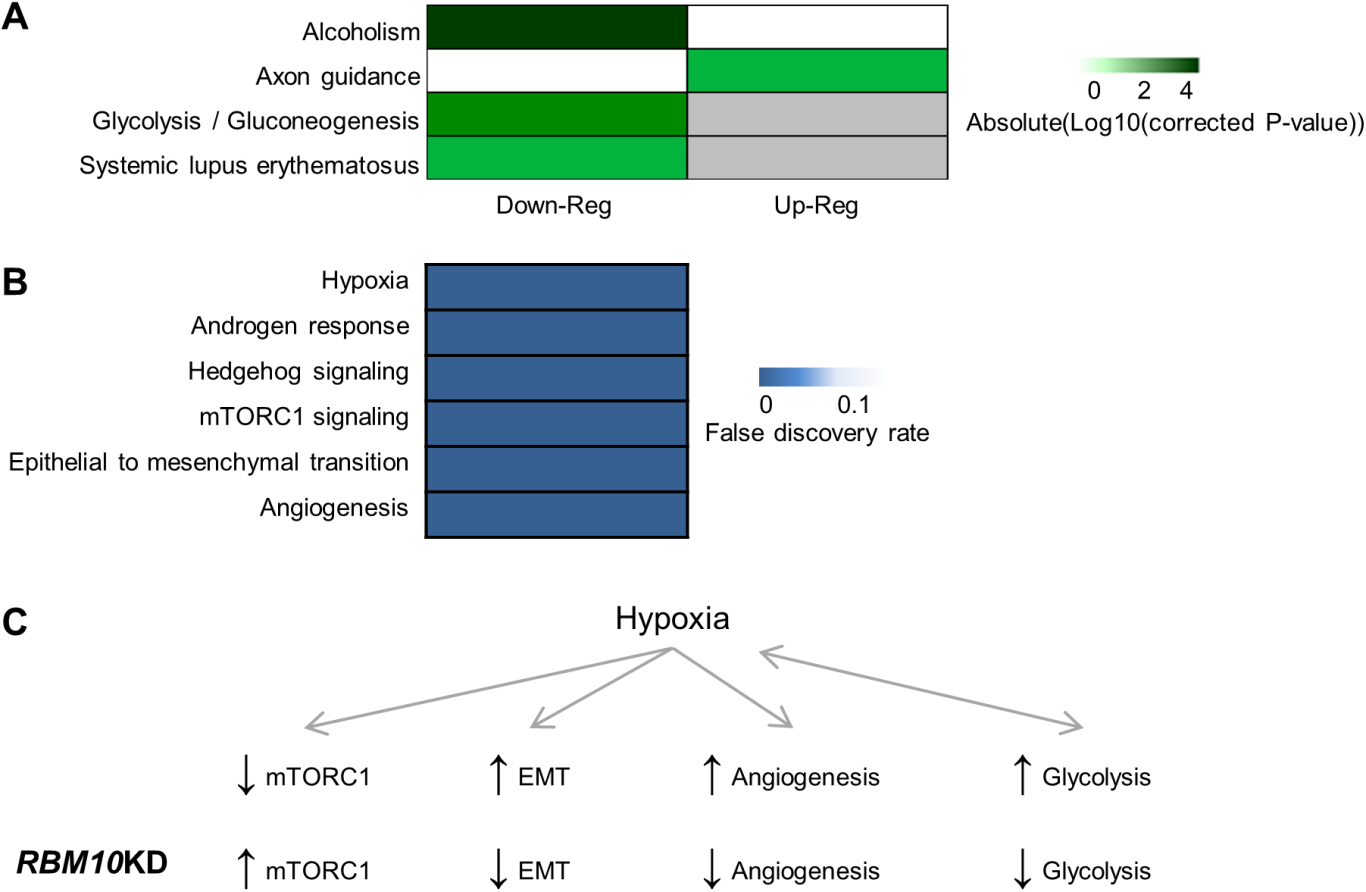
Pathway analysis of genes differentially expressed upon RBM10KD in GLC20 cells. (A) KEGG pathways significantly enriched upon RBM10KD in GLC20 cells. Analysis performed using subline RNA-Seq expression data and the FIDEA pathways analysis program. (B) MSigDB Hallmark gene sets enriched at a false discovery rate of below 5% upon RBM10KD in GLC20 cells. Analysis performed using subline RNA-Seq expression data and the GSAASeqSP pathway analysis program. (C) Relationship between select RBM10-altered pathways in GLC20 cells. Hypoxia is associated with decreased levels of mTORC1 signaling, and promotion of EMT and angiogenesis. In addition, hypoxia is associated with increased levels of glycolysis, and likewise, survival in hypoxic conditions is promoted by increased levels of glycolysis. The influence of RBM10KD on these pathways, as determined by RNA-Seq is indicated.

To determine the functional relevance of the differentially expressed genes, we performed pathway analyses. To ensure the validity of our results, as for the RIP-Seq data analyses, two different pathway analysis programs were used, each with their own database. We began with the FIDEA Program and the KEGG Database. With these tools, that group genes based on molecular interactions, we identified four enriched pathways (Fig 4A). The only upregulated pathway upon RBM10KD was ‘Axon guidance’. Genes involved in axon guidance are deleted or the subject of promoter hypermethylation in many cancers [39]. As *RBM10* expression would correlate with decreased expression of these ‘Axon guidance’ factors, this may be one way by which RBM10 could promote the transformed state of lung cells. On the other hand, all three pathways that were significantly downregulated upon RBM10KD are positively linked with cancer; (1) ‘Alcoholism’-related pathways have been shown to promote the transformed state, in part *via* increased EMT [40], (2) ‘Glycolysis’ is upregulated in cancers and is the main pathway for ATP production, even in the presence of oxygen [41], and (3) ‘Systemic lupus erythematosus’, an autoimmune disease, is linked with an increased risk of cancer, especially cancers of hematologic origin, as well as lung and hepatobiliary cancers [42, 43]. These results suggest that RBM10 could promote the transformed state in a wide variety of ways, including modulation of the immune system and various aspects of cell metabolism.

To complement our FIDEA and KEGG pathway analysis, we used the Gene Set Association Analysis with Sequence Permutation (GSAASeqSP) Program, which is specific for RNA-Seq data [44], with the Broad Institute’s Molecular Signatures Hallmark Database (MSigDB), which groups genes based on known functions [45]. As shown in Fig 4B, six gene sets with false discovery rates (FDR) below 5% were enriched upon RBM10KD in GLC20 cells. The most enriched gene set was ‘Hypoxia’, which coordinates well with the KEGG pathway analysis results described above; hypoxic conditions increase rates of glycolysis, and likewise, increased glycolysis supports tumor growth in hypoxic conditions [46]. Interestingly, three of the other five gene sets significantly enriched upon RBM10KD are also influenced by hypoxia; ‘mTORC1 signaling’, ‘Epithelial to Mesenchymal Transition’ (EMT) and ‘Angiogenesis’. Of note, the ‘mTORC1 signaling’ pathway, which regulates cell growth, is downregulated during hypoxia [47]. Conversely, EMT and angiogenesis can be promoted during hypoxia [48, 49] (Fig 4C). Although our GSAASeqSP analysis showed that these three hypoxia-related gene sets were enriched upon RBM10KD, we went on to determine if RBM10KD specifically promoted or inhibited these processes.

Firstly, in hypoxic conditions, downregulation of mTORC1 signaling is accomplished, in part, by ATM protein kinase, REDD1 and BNIP3 [47, 50, 51]. Interestingly, in our system, RBM10KD correlated with decreased levels of all three of these genes: *ATM* levels decreased from 27.98 fragments per kilobase of transcript per million mapped read (FPKM) (Control) to 20.188 FPKM (RBM10KD), REDD1 levels decreased from 71.65 FPKM (Control) to 30.96 FPKM (RBM10KD), and BNIP3 levels decreased from 58.02 FPKM (Control) to 19.70 FPKM (RBM10KD). These results suggest that mTORC1 signaling is promoted upon RBM10KD (Fig 4C). Secondly, we examined the impact of RBM10KD on the expression of previously published EMT-associated genes [52]. Notably, all genes listed as downregulated during EMT in the referenced review were significantly upregulated upon RBM10KD in GLC20 cells, or showed no significant change in expression. The significantly upregulated genes included *CDH1*, *TJP1*, *PATJ*, *JUP* and *DSC2*. On the other hand, all genes listed as upregulated during EMT, except fibronectin, were either significantly downregulated upon RBM10KD in GLC20 cells or showed no significant change expression. The significantly downregulated genes included *VIM*, *ID1* and *ZEB1*. Our results therefore show that RBM10KD is associated with decreased EMT (Fig 4C). This is in line with our KEGG pathway analysis, which showed that RBM10KD decreased the EMT-promoting ‘Alcoholism’ pathway.

Finally, we examined the expression of angiogenesis markers [49] in our RBM10KD samples: RBM10KD significantly decreased the expression of three by at least nine FPKM (*VEGF* from 20 to 9 FPKM; *FGF10* from 10 to 1 FPKM; *PLGF* from 75 to 33 FPKM), and increased the expression of two others, but only by 1–2 FPKM (*THBS1* from 1.75 to 3.8 FPKM; *ANGPT1* from 0.89 to 1.8 FPKM). These results show that, in general, RBM10KD correlates with decreased angiogenic marker expression (Fig 4C).

In summary, our GSAASeqSP pathway analysis showed that RBM10KD correlated with increased mTORC1 signaling, and decreased EMT and angiogenesis. Therefore, *RBM10* expression would be expected to downregulate mTORC1 signaling and promote EMT and angiogenesis, thereby influencing these processes in a similar way as hypoxia, and consequently promoting the transformed state. The large overlap in the results obtained using the two different pathway analysis programs (FIDEA and GSAASeqSP), each with their own database (KEGG and MSigDB), strongly supports our conclusion that RBM10 influences various hypoxia-related pathways to promote the transformed state. Together, our pathway analysis results suggest that, unlike RBM5, RBM10 promotes the transformed state. RBM10 does this specifically by (1) increasing glycolysis, (2) reducing mTORC1 signaling, which is associated with deregulated cell growth and could contribute to the higher viable cell metabolism activity observed in *RBM10* expressing GLC20 cells by MTT (Fig 3E), (3) promoting EMT, thereby contributing to the establishment and progression of the transformed state, and (4) promoting angiogenesis. This wide scope of influence is supported by the large number of RBM10-only targets identified by RIP-Seq.

### Differential gene expression in GLC20 RBM10KD cells compared to RBM5 expressing GLC20 cells

Having determined that RBM5 and RBM10 share similar targets in the C4 subline, and affect similar processes in SCLC, but with opposing functional consequences, we decided to identify and compare the genes influenced by both proteins. To this end, we examined differential gene expression following either RBM10KD or RBM5 expression in GLC20 cells. We anticipated that many of the same genes would be influenced, but in an opposite manner, by each protein.

**Fig 5:**
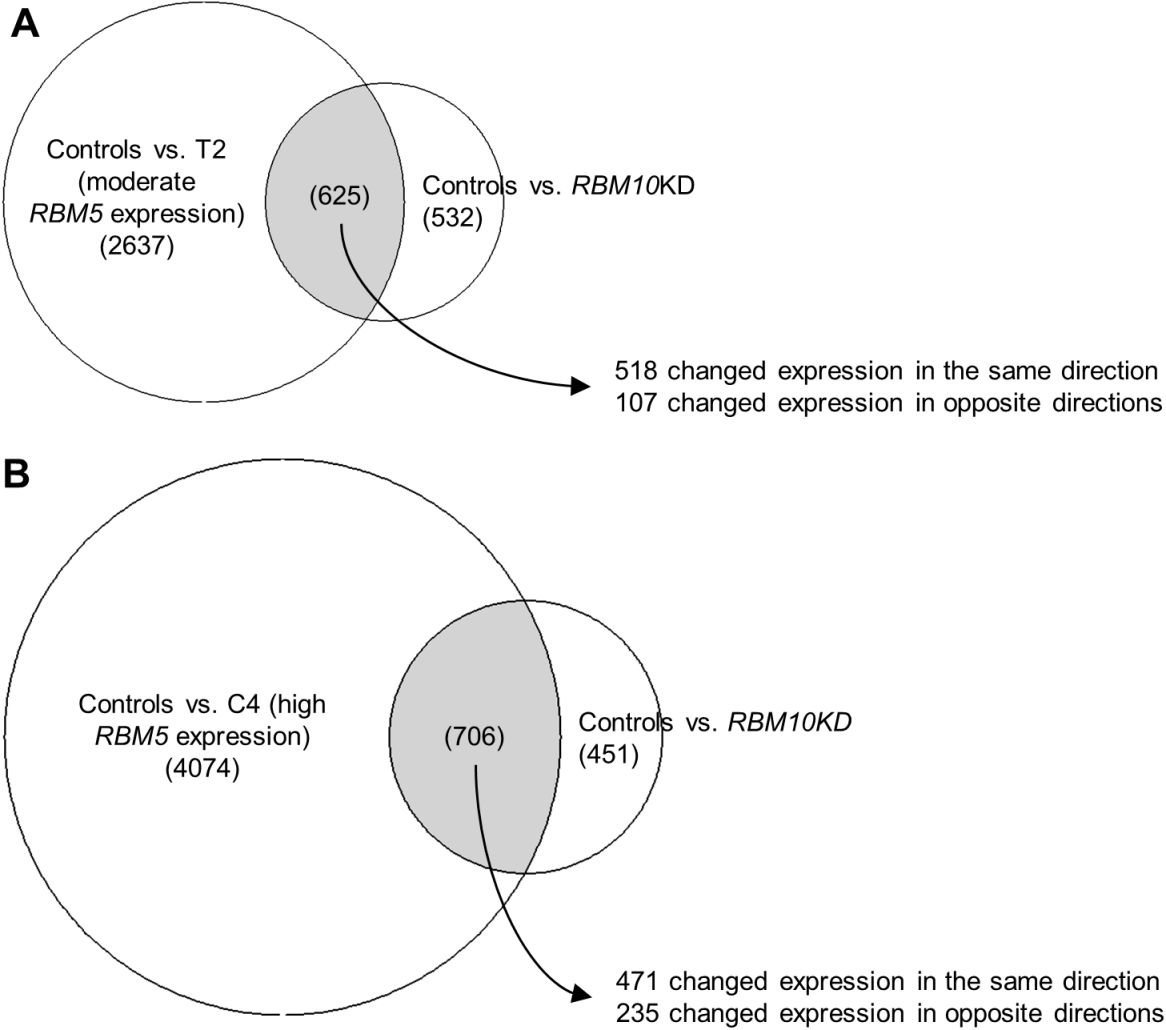
Comparison of genes differentially expressed upon RBM10KD in GLC20 cells or *RBM5* expression in GLC20 sublines. Differentially expressed genes, based on RNA-Seq results, in GLC20 cells upon RBM10KD, and moderate (A) or high (B) expression levels of *RBM5* expression.

Comparing the RNA-Seq data from our G29/30.4 GLC20 RBM10KD subline with the RNA-Seq data from the stable *RBM5*-expressing GLC20 sublines T2 and C4, we determined that many of the same genes were in fact differentially expressed following either RBM10KD or *RBM5* expression; of the significantly differentially expressed genes in RBM10KD, 53.8% were also significantly differentially expressed between Control and T2, and 60.7% were also differentially expressed between Control and C4 (Fig 5). As expected, well over half of these common differentially expressed genes (428 genes) were the same whether RBM10KD was compared to T2 or C4. Therefore, RBM5 and RBM10 do affect the expression of many of the same genes.

We then went on to determine how RBM5 and RBM10 levels affected the expression of these commonly differentially expressed genes. Between RBM10KD and T2 *RBM5* expression, 82% of the common significantly differentially expressed genes changed expression in the same direction (Fig 5A). As for RBM10KD and C4 *RBM5* expression, 67% of the differentially expressed genes changed expression in the same direction (Fig 5B). Next, we wanted to determine if this similarity would extend to genes specifically associated with SCLC. In June 2014, the National Cancer Institute compiled a list of ‘Genes of Interest in SCLC’, as part of their report entitled Scientific Framework for Small Cell Lung Cancer. We recently demonstrated that *RBM5* expression in C4 significantly altered the expression of 11 of these SCLC-associated genes in a way which would suppress tumor growth [9]. Here we now show that RBM10KD altered the expression of nine of these 11 genes in the same direction as *RBM5* expression; *BLC2*, *CCNE1*, *CREBBP*, *EPHA7*, *MED12L*, *MYCL*, *SLIT2*, *SMO* and *SOX2* (Table 2). This high level of similarity between the effect of *RBM5* expression and RBM10KD on gene expression, especially SCLC-associated genes, suggests opposing roles for RBM5 and RBM10 in aggressive SCLC, and supports our MTT and RNA-Seq findings.

**Table 2:** Expression of select National Cancer Institute (NCI) ‘Genes of Interest in SCLC’ in Control and RBM10KD samples, as determined by RNA-Seq.

Gene name	Control	RBM10KD	*p*-value
BCL2	1.81928	1.26218	0.29525
CCNE1	18.6741	15.4965	0.2551
COBL †	0.774161	1.82919	0.00095
CREBBP ‡	5.54439	9.8752	0.0001
EPHA7	16.5578	21.467	0.09965
MED12L †	4.10916	2.75174	0.0181
MYCL	11.3944	8.92857	0.17185
RAB37	0.737284	0.803781	0.758
SLIT2 ‡	5.30312	10.1706	0.00005
SMO †	5.8898	4.02038	0.04565
SOX2 †	13.317	8.46985	0.0124

† Genes significantly downregulated upon RBM10KD (*p* < 0.05).

‡ Genes significantly upregulated upon RBM10KD (*p* < 0.05).

### Effect of RBM5 on RBM10 expression

Previous studies have shown tumor-suppressor properties for both RBM5 and RBM10. In the results presented herein, however, using an aggressive *RBM5*-null SCLC model, we show an opposite effect for RBM10, correlating its expression with promotion of various transformation-associated processes. These results suggested to us that RBM5 may be a regulator of RBM10 function, a relationship previously supported by our work in a rat myoblast system [20]. To investigate if and how RBM5 might influence RBM10, we examined *RBM10* mRNA and protein expression in our stable *RBM5*-expressing GLC20 sublines T2 and C4.

**Fig 6:**
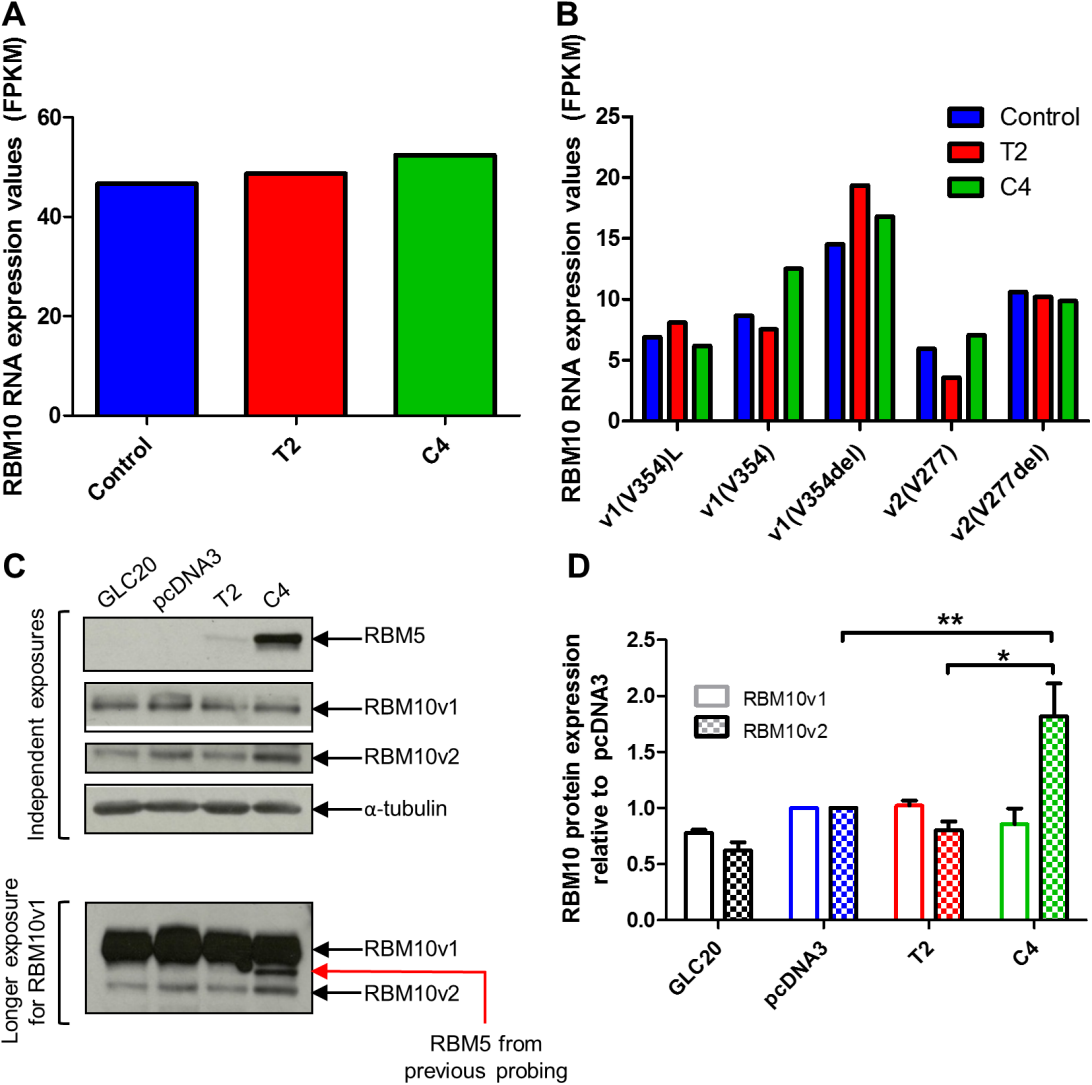
*RBM10* expression in the parental GLC20 cell line and stable *RBM5*-expressing sublines. (A) Expression of all *RBM10* mRNA variants, as determined by RNA-Seq. (B) mRNA expression of specific *RBM10* splice variants, as determined by RNA-Seq. ‘Control’ in A and B refers to the GLC20 parental cell line and stable pcDNA3-transfected GLC20 subline. (C) Representative Western blot for RBM5 and RBM10 (Sigma antibody) protein expression levels. Alpha-tubulin was used as loading control. (D) Densitometric results of the average RBM10v1/v2 expression of three biological replicates each performed in technical duplicate. Analysis was performed using AlphaEaseFC, ‘1D-Multi’ analysis tool. Values of RBM10v1 and RBM10v2 were normalized to the α-tubulin of each biological technical duplicate, and then made relative to the pcDNA3 controls. Standard error is presented. One-way ANOVA was performed with Tukey post-hoc analysis, between sublines. * *p* < 0.05 and ** *p* < 0.01.

*RBM10* mRNA levels in T2 and C4 were determined from RNA-Seq data [9]. Since *RBM10* splice variants differ, in some cases, by only one amino acid, this level of resolution was required to precisely quantify the expression of each variant (Fig 1). Differential expression analysis of RNA-Seq results showed no significant change in *RBM10* mRNA expression levels following *RBM5* expression in GLC20 cells (Fig 6A). There was also no significant change in the expression of any *RBM10* splice variant when RBM5 levels were altered (Fig 6B). RBM5 did not, therefore, influence *RBM10* transcription or alternative splicing.

To determine if RBM5 is capable of influencing *RBM10* post-transcriptionally, RBM10 protein levels were investigated *via* Western blot. To note, only the two major isoforms of RBM10 (v1 and v2) are distinguishable on Western blots. Interestingly, RBM10v1 levels were not altered between sublines, whereas RBM10v2 levels were significantly increased in C4 compared to either T2 or the Control (Fig 6C and 6D). These results demonstrated that RBM5 was capable of influencing *RBM10* expression, that this influence occurred post-transcriptionally, and that the effect was restricted to *RBM10v2*.

**Fig 7:**
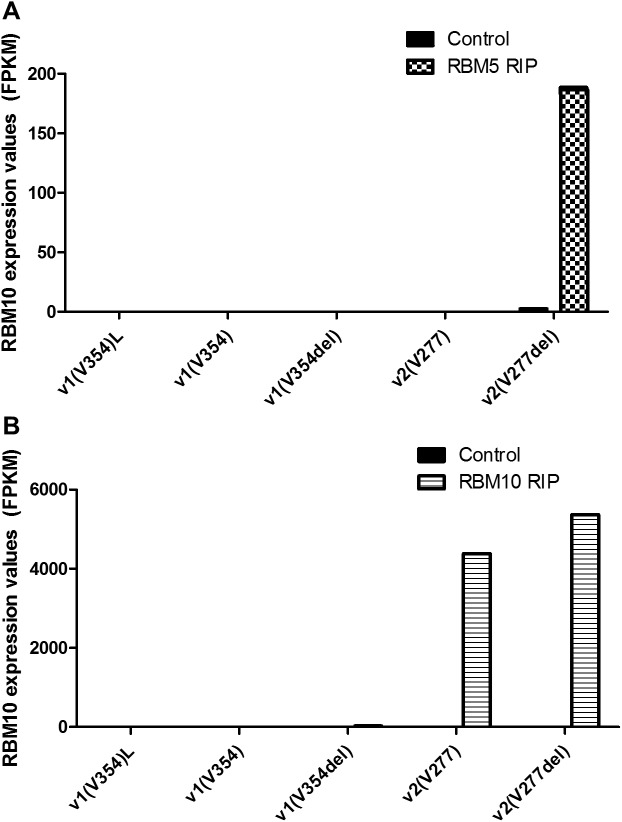
RBM5 and RBM10 RIP-Seq results for *RBM10* splice variants. Graph representing the expression of the various *RBM10* splice variants in RBM5 (A) and RBM10 (B) RIP-Seq experiments, which were carried out in C4 cells.

This increase in RBM10v2 levels could potentially have resulted from a targeted increase of *RBM10v2* translation, or from stabilization of the RBM10v2 isoform, by high levels of RBM5. Increased *RBM10v2* translation would likely involve binding of RBM5 protein to *RBM10v2* transcript, whereas stabilization of the RBM10v2 isoform by RBM5 would involve a protein/protein interaction. As a next step in our investigations, we decided to examine the ability of RBM5 protein to interact with *RBM10* transcripts. Towards this end, we re-examined our previous RBM5 RIP-Seq data (from C4 cells) [9]. As RIP-Seq provides a single nucleotide resolution of all RNA targets, we would be able to determine if RBM5 interacted with specific *RBM10* splice variants. As shown in Fig 7A, RBM5 bound only the one specific *RBM10* splice variant, *RBM10v2(V277del)*. RBM5 bound 186.357 FPKM of *RBM10v2(V277del)*, compared to 2.62 FPKM of IgG bound *RBM10v2(V277del)* in the Control RIP. All other RBM10 splice variants had counts of 0.1 FPKM or below, in the RBM5 RIP sample, a result clearly unrelated to variant expression levels in C4 (Fig 6B). This level of specificity, in regards to RBM5’s binding of only one RBM10 splice variant, is likely due to structural differences between RBM10 alternative splice transcripts, which is supported by our preliminary RNA structure analysis (S1 Fig) [53]. Interestingly, in an RBM5-null environment, we found that RBM10 was also capable of binding self; specifically, using our RIP-Seq data, we found that just like RBM5, RBM10 bound *RBM10v2(V277del)* mRNA. Unlike RBM5, which was only able to bind the one *RBM10v2* variant, RBM10 was able to bind not only both variants of *RBM10v2*, at high levels (Fig 7B), but also *RBM10v1(V354del)*, to a lesser extent (28.1 FPKM). These results show that RBM10 protein can in fact bind *RBM10* mRNA and thus it may, like RBM5, be involved in regulating RBM10 isoform expression.

## Discussion

Functional RBM10 is important for normal cellular processes. For instance, mutations in *RBM10* are the cause of TARP syndrome, which results in many developmental abnormalities and often lethality before or soon after birth [11, 54, 55]. Furthermore, *RBM10* is mutated in pancreatic intraductal papillary mucinous neoplasms [56], 7% of lung adenocarcinomas [57], and 21% of invasive lung adenocarcinomas [21]. *RBM10* has also been suggested to be important to rat myoblast differentiation [20]. Therefore, understanding the regulation of RBM10, as well as the range of its downstream effects, is of interest to various areas of cell, developmental and cancer biology.

Previous studies relating to RBM10, which have been performed in *RBM5*-expressing systems, have primarily linked *RBM10* expression with reduced cell proliferation and promotion of apoptosis [10, 14–17]. The study reported here explores, for the first time, the function of RBM10 in an *RBM5*-null system, and the regulation of *RBM10* by RBM5. As *RBM5* is downregulated in many cancers [8, 58–62], this work is particularly meaningful. Here we show that RBM10 actually promotes transformation-associated processes in an *RBM5*-null environment. Supporting our findings, *RBM10* has been associated with increased expression of VEGF, a promoter of new blood vessel growth, in breast cancer samples [16]. In addition, in patients with metastatic melanoma, high *RBM10* expression was correlated with increased disease aggression [19]. Of note, the *RBM5* promoter was identified as a “Signature of accelerated somatic evolution” in melanomas, with mutations in this region correlating with significantly lower survival rates and a higher incidence of metastasis [63]. Therefore, altered RBM5 levels in melanoma may result in decreased translational regulation of RBM10, and thus the pro-transformation characteristics associated with RBM10 in melanomas, and here in our *RBM5*-null system. In fact, in solid tumors in general, *RBM5* is one of nine genes, within a 17 gene signature associated with metastasis, that are downregulated in various humans [64] and mice [65]. Thus, the increased metastatic characteristics of tumors with decreased RBM5 levels could in part involve decreased translational regulation of RBM10 by RBM5. This is particularly relevant as we show that RBM10 upregulates processes such as EMT and angiogenesis in an *RBM5*-null system. The majority of functional studies relating to RBM10 have focused on its role in modulation of alternative splicing [14, 17, 66–69], likely due to the early findings that RBM10 is a core component of spliceosomal A and B complexes [25, 26, 70]. Our RNA-Seq results show, however, no significant shifts in alternative splicing upon RBM10KD in GLC20 cells (no genes had a significant increase in the expression of one alternative splice variant and a significant decrease in the expression of another) (S2 Table), a finding we postulate may be dependent on RBM5 expression. Of note, upon RBM5 expression in GLC20 cells, using the same techniques and analysis parameters, we detected multiple alternative splicing changes. Our transcriptomic analyses, which show no effect of RBM10 downregulation on alternative splicing in GLC20 cells, are supported by (1) our RBM10-only targets (Table 1), which are involved in various aspects of gene expression regulation, excluding alternative splicing, (2) RBM10’s mRNA stabilization effect on its only identified direct RNA target, the AT1 receptor [71], and (3) RBM10’s interaction with the 2A-DUB deubiquitinase protein complex [72] and the Rac-specific GTPase-activitating protein FilGAP [73]. These findings are not unanticipated considering RNA binding proteins are known to participate in multiple aspects of RNA metabolism, including RNA editing, polyadenylation, export, localization, translation and stability [74]. Future studies regarding RBM10 should therefore attempt to capture its influence on these other aspects of RNA biology, and not be restricted to alternative splicing. Falling to do so would result in an incomplete view of the scope of RBM10’s influence on the cell.

More important than the role of RBM10 in the regulation of alternative splicing might be the role of alternative splicing of *RBM10* itself. The single valine residue that differentiates the two *RBM10v1* variants, as well as the two *RBM10v2* variants, occurs within the second RNA Recognition Motif (RRM) of RBM10. The presence or absence of this valine residue can influence the α-helical structure of this RRM domain, and could thus influence RBM10’s binding targets [13, 14]. Although the functional implications of this modification have yet to be tested, the level of binding specificity exhibited by RBM5 suggests that (1) the presence or absence of this valine residue modifies RBM10’s structure sufficiently for proteins (at least RBM5) to be able to distinguish between variants, and (2) *RBM10* splice variants have specific, potentially opposing, roles in the cell, and thus require specific regulation.

Based on our results and those of previous RBM10-related studies, we propose a working model describing RBM5’s influence on *RBM10* and yet to be confirmed functional interactions between RBM5 and RBM10 (S2 Fig). This is an extension of a previous model we developed in a non-transformed rat myoblast system, which described how RBM10 might influence RBM5 [20]. We hypothesize that RBM10’s binding to *RBM10v2* mRNA results in a downregulation of RBM10v2 protein expression levels. This would explain the much lower levels of RBM10v2 protein, compared to RBM10v1, that we observed in all GLC20 sublines (Fig 6C), even though mRNA levels for both variants were very similar (Fig 6A and 6B). Furthermore, we suggest that RBM5’s binding to *RBM10v2(V277del)* promotes this transcript’s translation or prevents its degradation, resulting in the higher levels of RBM10v2 protein seen in the C4 population. The lack of significantly higher RBM10v2 protein expression in T2 (Fig 6C), compared to Control, is not unexpected since this subline has substantially less RBM5 expressed than C4. We postulate that RBM10v2 is an essential component, or participates in the formation, of certain RBM5 protein complexes, which would explain why (1) RBM5 would upregulate RBM10v2 protein levels, (2) such a large proportion of RBM5 targets were also shared by RBM10 in our RIP-Seq experiments, and (3) RBM10 would have so many RBM10-only targets; these targets would represent RBM10v1 targets, which would be independent from those targeted by the RBM5-RBM10v2 complex.

We propose that the RBM5-RBM10v2 complex regulates cell cycle progression and promotes apoptosis, functions with which RBM5 has been previously associated. We suggest that most, but not all, RBM5’s tumor-suppressor properties occur *via* this RBM5-RBM10v2 complex. The RBM5-RBM10v2 complex could also be responsible for the alternative splicing events previously associated with RBM10. This would explain why, in an *RBM5*-null system, these alternative splicing events do not appear to be influenced by RBM10 (*e*.*g*., there would be no RBM5-RBM10v2 complex). On the other hand, we suggest that RBM10v1 promotes transformation-associated processes, including glycolysis, EMT and angiogenesis. Some of the downstream RBM5-RBM10v2 and RBM10v1 targets overlap, with both complexes affecting the expression of these genes in an opposing manner. This would explain the similarity of differentially expressed genes upon *RBM5* expression and RBM10KD in GLC20 cells, but why RBM5 and RBM10 affected the expression of these genes in contrasting ways (Fig 5). We also propose that the RBM5-RBM10v2 complex has a more significant influence on transformation-associated pathways than RBM10v1, explaining why cells expressing physiological levels of both RBM5 and RBM10 have regulated cell growth (as seen in the T2 subline) [9].

According to our working model, upon *RBM5* expression, an RBM5-RBM10v2 complex would be formed, resulting in higher levels of apoptosis and cell cycle arrest (S3A Fig). This is in line with what we have previously shown experimentally upon *RBM5* expression in GLC20 cells [9]. Inversely, upon *RBM5*KD, less RBM5-RBM10v2 complex would be formed, resulting in decreased apoptosis and deregulated cell growth, as well as less competition for RBM10v1 regarding their common targets (S3B Fig). This would all contribute to promotion of a transformation-like phenotype. Indeed, this is what we observed previously in parental GLC20 cells, compared to the *RBM5*-expressing sublines [9]. Upon RBM10KD in an *RBM5*-expressing system, we would expect almost no RBM5-RBM10v2 complex to be formed, especially as RBM10v2 levels are already so much lower than RBM10v1 in GLC20 cells (S3C Fig). This would result in a significant decrease in apoptosis, as well as reduced regulation of cell cycle progression. Although knockdown of *RBM10* would also significantly decrease RBM10v1 levels, since RBM10 would not be completely knocked out in this situation, some RBM10v1 would remain in the cell. This small amount of RBM10v1 could still promote transformation-associated processes to a certain point, especially with much less competition from the RBM5-RBM10v2 complex. In this case, we would expect a slight increase in cell growth upon RBM10KD in *RBM5*-expressing systems. This hypothesis is in line with previous functional work performed for RBM10 in endogenously *RBM5*-expressing HeLa cells [17]. Finally, upon RBM10KD in an *RBM5*-null system, since there would be no influence from the RBM5-RBM10v2 complex, RBM10KD would only significantly reduce levels of RBM10v1 (S3D Fig). This would result in diminished promotion of transformation-associated processes, and thus lower levels of cell growth and/or metabolism. This proposition is in line with the MTT and RNA-Seq results presented herein regarding RBM10KD in endogenously *RBM5*-null GLC20 cells (Figs 3E and 4).

Interestingly, *RBM10* mutations have recently been associated with lung adenocarcinoma pathogenesis [66]. Loss-of-function *RBM10* mutations would result in no functional RBM10 in the cell and, based on our working model, no RBM5-RBM10v2 complex. This would have two opposing consequences; (1) loss of RBM10v1 would result in reduced promotion of transformation-associated processes, and (2) loss of RBM5-RBM10v2 complex would result in reduced regulation of the cell cycle and decreased apoptosis, and thus promotion of transformation. Since, as we proposed above, the influence of RBM5-RBM10v2 is greater than RBM10v1, we would expect the overall effect of loss-of-function *RBM10* mutations to be pro-transformatory, similar to the effects of RBM5KD. This is indeed the result seen in clinical samples; loss-of-function *RBM10* mutations were associated with increased pathogenesis of lung adenocarcinomas [21]. These findings further support our working model.

A very recent study in non-transformed mouse cells not only supports our working model, but extends its relevance beyond cancer-based systems [75]. In that study, Rodor *et al*. knocked out *RBM10* in mouse cells and examined a number of processes, including cell proliferation. According to our working model, since RBM10 would be completely depleted in this scenario, RBM10v1 could no longer promote transformation-associated processes and RBM5-RBM10v2 complexes would be completely eliminated. Since RBM10 would no longer influence cellular processes, RBM5 could exercise its RBM10v2-independent tumor-suppressor properties. We would thus predict that RBM10 knockout would result in a decrease in cell proliferation. This is indeed what the authors found. Although Rodor *et al*. strongly associated RBM10 with the regulation of alternative splicing in that study [75], as mouse cells in general express RBM5 [76], this could be an RBM5-dependant function of RBM10, as we propose above.

Considered together, our results suggest that the RBM10 reversal-of-function associated with downregulation of *RBM5* is one means by which cells transition to a cancerous state, largely through processes that involve regulation of a hypoxic state. Our results shed light on the relationship between RBM5 and RBM10, a particularly important aspect to remember when studying either of these genes individually. We not only determine, for the first time, the role of RBM10 in an *RBM5*-null system, but also propose a working model to explain how RBM10 is influenced by *RBM5* expression. We also identify all RBM10 targets and explore the extent of RBM10’s influence on cellular processes. In addition, we show that RBM5 directly binds *RBM10* to regulate its expression post-transcriptionally, and that RBM5 and RBM10 share similar direct (RIP-Seq) and downstream (RNA-Seq) targets. These results lay the groundwork for additional studies to examine the role(s) of specific RBM10 isoforms, and the relationship between RBM5 and RBM10 in different systems and disease states.

## Supporting information

S1 Fig Predicted mRNA structure of RBM10 splice variants. Most probable RNA structure for (A) RBM10v1(V354), (B) RBM10v1(V354del), (C) RBM10v2(V277) and (D) RBM10v2(V277del), as determined by RNAstructure MaxExpect using standard settings. Structure generated is composed of highly probable base pairs. Black arrow indicates position of the ‘GTG’ codon alternatively spliced from both RBM10v1 and RBM10v2, which results in their respective +/- valine isoforms.
(PPTX)

S2 Fig Working model representing RBM5 and RBM10 function and interaction in SCLC. Rectangles represent mRNA, whereas ovals represent protein. Blue ovals are unspecified proteins.
(PPTX)

S3 Fig Prediction of the effects of modulating RBM5 and/or RBM10 levels. Using our working model, we predict the effects of (A) RBM5 overexpression, (B) RBM5KD, (C) RBM10KD in an RBM5-expressing system, and (D) RBM10KD in an RBM5-null system. Open green, blue and purple arrows indicate effect, and thickness of said arrows indicates activity levels (dotted arrows indicate least activity). Closed red arrows indicate direction of expression change only. For detailed description of model, refer to text.
(PPTX)

S1 Table RBM5 and RBM10 RIP-Seq results. RNA expression levels of all genes examined in RBM5 and RBM10 RIP-Seq experiments. Gene expression quantification was performed using Cuffdiff v2.2.1; however, targets were identified using the inclusion criteria elaborated in the Materials and Methods section. Genes listed based on log2(fold-change) in expression, and then their value in the experimental RIP sample.
(XLSX)

S2 Table RNA-Seq gene expression data for control and RBM10KD GLC20 cells. RNA expression levels for all genes examined, as well as their specific splice variants. Genes listed based on their q-value, and log2(fold-change) in expression. Ctrl refers to combined gene expression values for parental GLC20 cells and the stable pcDNA3 transfected GLC20 subline. Differential expression testing was performed using Cuffdiff v2.2.1. The third sheet contains the information required to identify a particular gene’s alternative splice variant(s) (based on their TCONS assigned number from Cufflinks).
(XLSX)
