# Supplementary material for: RBM10 promotes transformation-associated processes in small cell lung cancer and is directly regulated by RBM5
Source: PLoS One. 2017 Jun 29;12(6):e0180258. doi: 10.1371/journal.pone.0180258 (PMC5491171; doi:10.1371/journal.pone.0180258)
Supplement: S1 Fig — Most probable RNA structure for (A) RBM10v1(V354), (B) RBM10v1(V354del), (C) RBM10v2(V277) and (D) RBM10v2(V277del), as determined by RNAstructure MaxExpect using standard settings. Structure generated is composed of highly probable base pairs. Black arrow indicates position of the ‘GTG’ codon alternatively spliced from both RBM10v1 and RBM10v2, which results in their respective +/- valine isoforms. (PPTX) [file pone.0180258.s001.pptx]

## Slide 1
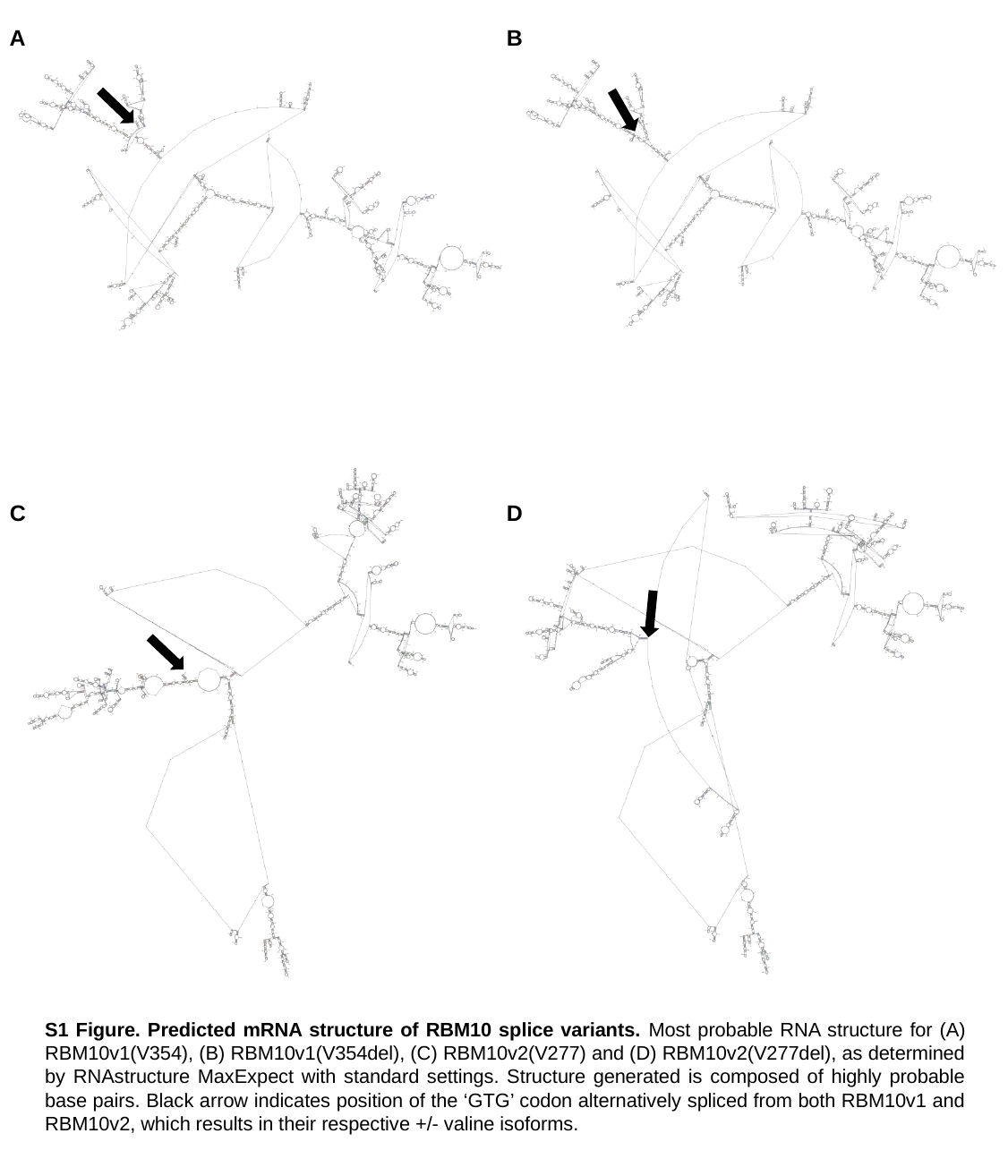

A
B
C
D
S1 Figure. Predicted mRNA structure of RBM10 splice variants. Most probable RNA structure for (A) RBM10v1(V354), (B) RBM10v1(V354del), (C) RBM10v2(V277) and (D) RBM10v2(V277del), as determined by RNAstructure MaxExpect with standard settings. Structure generated is composed of highly probable base pairs. Black arrow indicates position of the ‘GTG’ codon alternatively spliced from both RBM10v1 and RBM10v2, which results in their respective +/- valine isoforms.
