# Supplementary material for: RBM10 promotes transformation-associated processes in small cell lung cancer and is directly regulated by RBM5
Source: PLoS One. 2017 Jun 29;12(6):e0180258. doi: 10.1371/journal.pone.0180258 (PMC5491171; doi:10.1371/journal.pone.0180258)
Supplement: S2 Fig — Rectangles represent mRNA, whereas ovals represent protein. Blue ovals are unspecified proteins. (PPTX) [file pone.0180258.s002.pptx]

## Slide 1
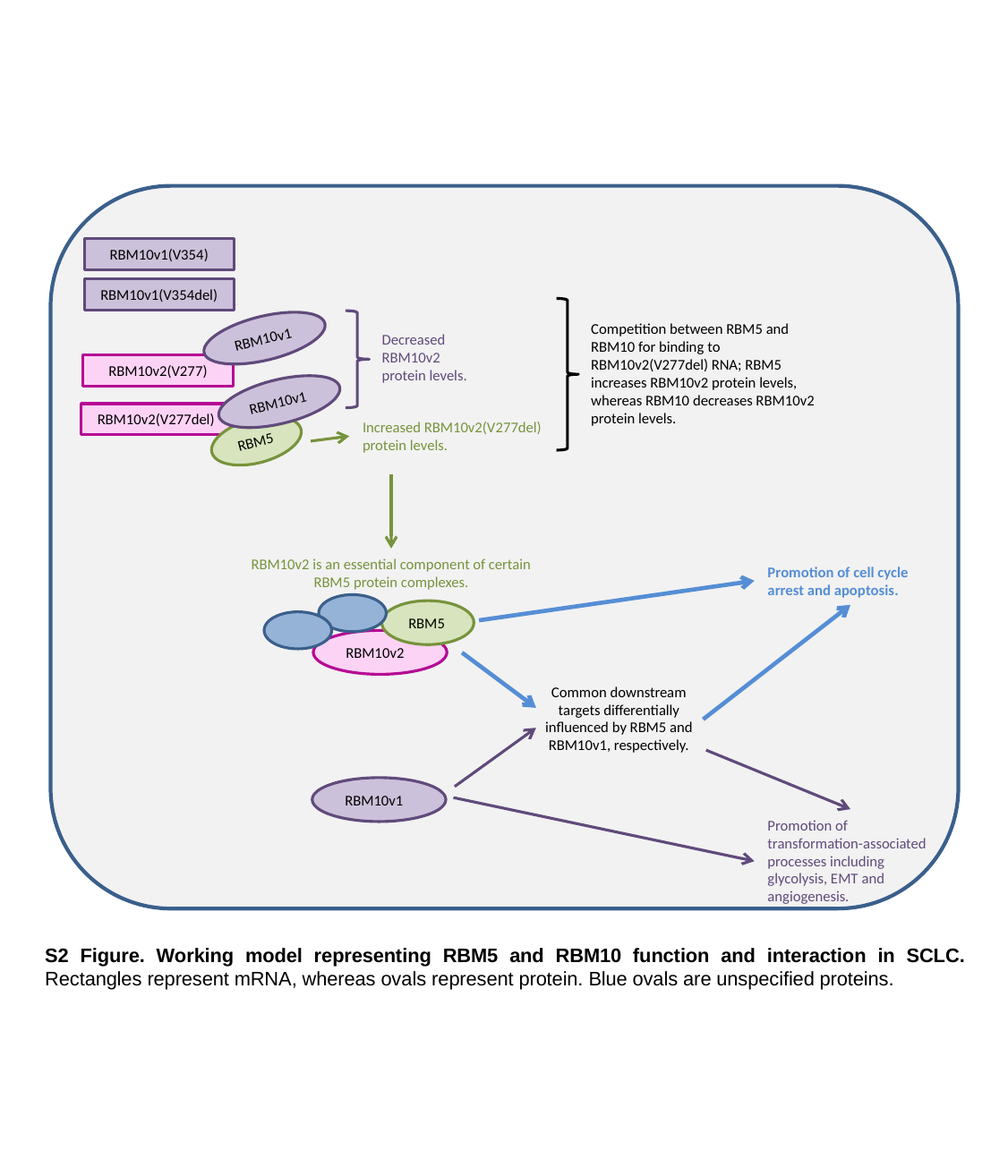

RBM10v1(V354)
RBM10v1(V354del)
Competition between RBM5 and RBM10 for binding to RBM10v2(V277del) RNA; RBM5 increases RBM10v2 protein levels, whereas RBM10 decreases RBM10v2 protein levels.
RBM10v1
Decreased RBM10v2 protein levels.
RBM10v2(V277)
RBM10v1
RBM10v2(V277del)
Increased RBM10v2(V277del) protein levels.
RBM5
RBM10v2 is an essential component of certain RBM5 protein complexes.
Promotion of cell cycle arrest and apoptosis.
RBM5
RBM10v2
Common downstream targets differentially influenced by RBM5 and RBM10v1, respectively.
RBM10v1
Promotion of transformation-associated processes including glycolysis, EMT and angiogenesis.
S2 Figure. Working model representing RBM5 and RBM10 function and interaction in SCLC. Rectangles represent mRNA, whereas ovals represent protein. Blue ovals are unspecified proteins.
