# Supplementary material for: RBM10 promotes transformation-associated processes in small cell lung cancer and is directly regulated by RBM5
Source: PLoS One. 2017 Jun 29;12(6):e0180258. doi: 10.1371/journal.pone.0180258 (PMC5491171; doi:10.1371/journal.pone.0180258)
Supplement: S3 Fig — Using our working model, we predict the effects of (A) RBM5 overexpression, (B) RBM5KD, (C) RBM10KD in an RBM5-expressing system, and (D) RBM10KD in an RBM5-null system. Open green, blue and purple arrows indicate effect, and thickness of said arrows indicates activity levels (dotted arrows indicate least activity). Closed red arrows indicate direction of expression change only. For detailed description of model, refer to text. (PPTX) [file pone.0180258.s003.pptx]

## Slide 1
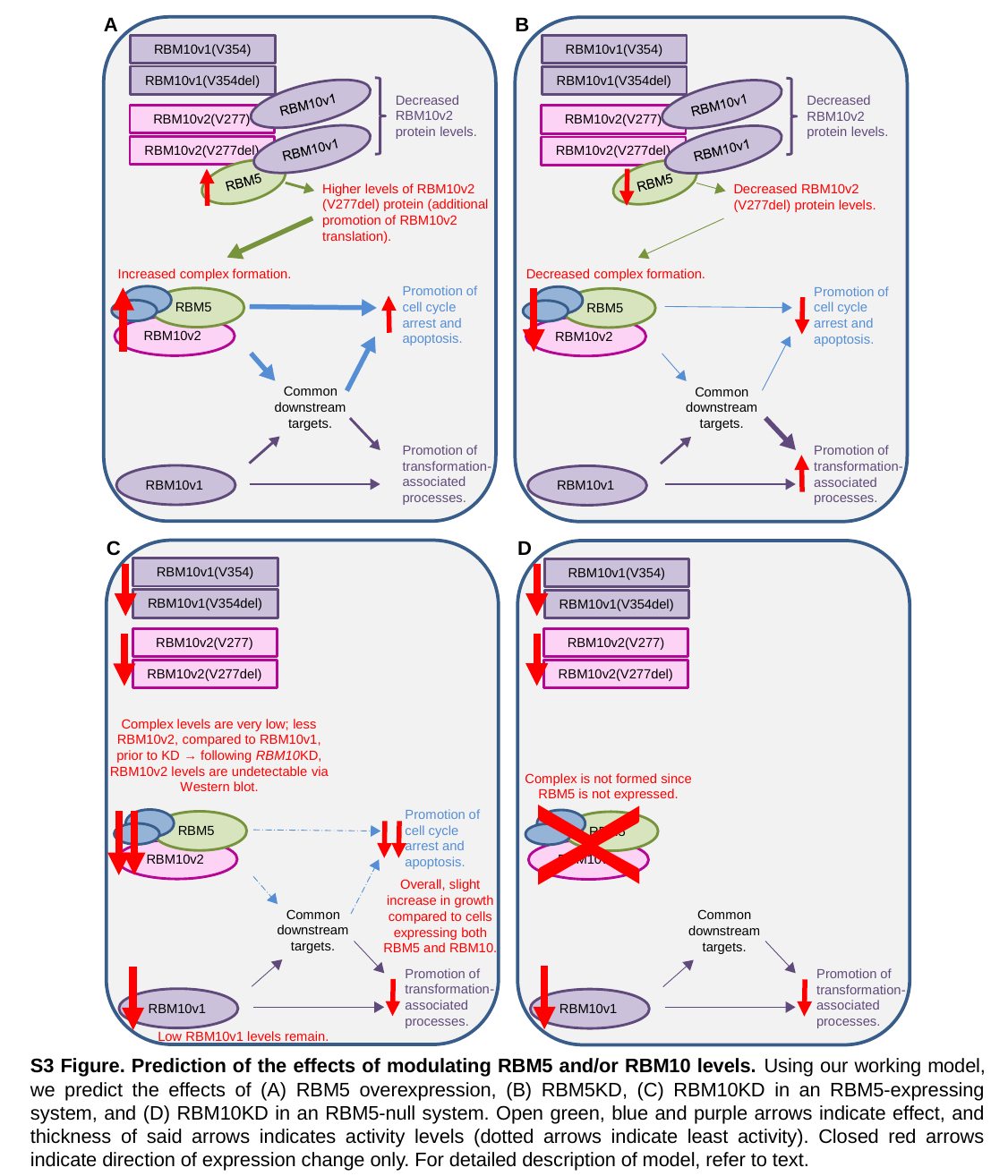

S3 Figure. Prediction of the effects of modulating RBM5 and/or RBM10 levels. Using our working model, we predict the effects of (A) RBM5 overexpression, (B) RBM5KD, (C) RBM10KD in an RBM5-expressing system, and (D) RBM10KD in an RBM5-null system. Open green, blue and purple arrows indicate effect, and thickness of said arrows indicates activity levels (dotted arrows indicate least activity). Closed red arrows indicate direction of expression change only. For detailed description of model, refer to text.
